# Reduced transient receptor potential vanilloid 2 expression in alveolar macrophages causes COPD in mice through impaired phagocytic activity

**DOI:** 10.1186/s12890-019-0821-y

**Published:** 2019-03-26

**Authors:** Hiroaki Masubuchi, Manabu Ueno, Toshitaka Maeno, Koichi Yamaguchi, Kenichiro Hara, Hiroaki Sunaga, Hiroki Matsui, Masahiro Nagasawa, Itaru Kojima, Yuko Iwata, Shigeo Wakabayashi, Masahiko Kurabayashi

**Affiliations:** 1 Department of Allergy and Respiratory Medicine, Gunma University Graduate School of Medicine, 3-39-22 Showa-machi, Maebashi, Gunma 371-8511 Japan; 2 Department of Cardiovascular Medicine, Gunma University Graduate School of Medicine, 3-39-22 Showa-machi, Maebashi, Gunma 371-8511 Japan; 3 Department of Laboratory Sciences, Gunma University Graduate School of Health Sciences, 3-39-22 Showa-machi, Maebashi, 371-8511 Gunma Japan; 4 Institute for Molecular and Cellular Regulation, Gunma University, 3-39-15 Showa-machi, Maebashi, Gunma 371-8512 Japan; 5 Department of Molecular Physiology, National Cerebral and Cardiovascular Center Research Institute, 5-7-1 Fujisirodai, Suita, Osaka, 565-8565 Japan; 6 Department of Pharmacology, Osaka Medical College, 2-7 Daigaku-machi, Takatsuki-city, 569-8686 Osaka Japan

**Keywords:** Alveolar macrophage, Chronic obstructive pulmonary disease (COPD), Cigarette smoke, Phagocytosis, Transient receptor potential V2 (TRPV2)

## Abstract

**Background:**

Defective phagocytosis in alveolar macrophages is associated with chronic obstructive pulmonary disease (COPD). Transient receptor potential cation channel subfamily V member 2 (TRPV2), a type of nonselective cation channel pertinent to diverse physiological functions, regulates macrophage phagocytosis. However, the role of TRPV2 in COPD remains poorly understood. Here, we explored the role of TRPV2 in the development of COPD.

**Methods:**

Macrophage TRPV2 expression and phagocytosis function were measured in MH-S cells (a murine alveolar macrophage cell line) and a cigarette smoke exposure mouse model.

**Results:**

TRPV2 expression and phagocytosis function were reduced when MH-S cells were exposed to cigarette smoke extract (CSE). TRPV2 knockdown by siRNA decreased phagocytosis in MH-S cells. Consistently, TRPV2 expression was reduced in alveolar macrophages prepared from bronchoalveolar lavage samples of mice which were exposed to cigarette smoke for 2 months. In addition, the alveolar space was progressively enlarged during development in TRPV2 knockout (TRPV2KO) mice. Moreover, exposure to cigarette smoke for 2 months significantly induced alveolar space enlargement in TRPV2KO mice, but not in wild-type (WT) mice. The phagocytic function of alveolar macrophages from TRPV2KO mice was reduced, compared with macrophages from WT mice.

**Conclusions:**

TRPV2 expression is profoundly downregulated in alveolar macrophages at early time points of cigarette smoke exposure. Reduced TRPV2-mediated phagocytic function renders the lung susceptible to cigarette smoke-induced alveolar space enlargement. TRPV2 may provide a therapeutic target for COPD induced by cigarette smoke.

**Electronic supplementary material:**

The online version of this article (10.1186/s12890-019-0821-y) contains supplementary material, which is available to authorized users.

## Background

Chronic obstructive pulmonary disease (COPD) is defined as small airway obstruction that is typically progressive, and is attributed to long-term exposure to toxic gases and particles, principally in relation to cigarette smoking. There is an increased number of alveolar macrophages in lung tissue from COPD patients [1], which is directly associated with the degree of COPD [2, 3]. Among the mechanisms relevant to COPD and emphysema development are chronic inflammation, protease-antiprotease imbalance, endoplasmic reticulum stress, accelerated senescence, and oxidative stress.

Alveolar macrophages act to maintain homeostasis of small airways by engulfing apoptotic cells and pathogens, as well as by releasing a variety of cytokines, chemokines, and proteases that regulate airway structure and function [4–8]. Previous studies have provided evidence for defective phagocytic function of alveolar macrophages in COPD [9–12]. Alveolar macrophages from COPD patients are defective in their function to engulf apoptotic bronchial epithelial cells and invading bacteria, despite smoking cessation [13]. COPD patients have increased susceptibility to pneumococcal pneumonia, at least partly because of defective phagocytosis by alveolar macrophages as a result of smoke exposure [12]. Thus far, numerous molecules have been identified as possible contributors to this altered alveolar macrophage phagocytosis, but the precise mechanisms remain unknown.

Transient receptor potential cation channel subfamily V member 2 (TRPV) channels are nonselective cation channels that have diverse physiological functions [14]. Multiple TRPV channel subtypes have been identified in a variety of tissues [15–17]. Recently, TRPV2 expressed in the alveolar macrophage cell membrane was revealed to mediate the earliest steps of macrophage phagocytosis [18, 19]. Upon exposure to phagocytic substrates, TRPV2 in macrophages is recruited to the nascent phagosome and evokes depolarization of the plasma membrane, which then activates signalling pathways leading to the actin polymerization necessary for phagocytic receptor clustering. TRPV2-deficient macrophages are defective in chemoattractant-elicited motility after pathogen challenge, and TRPV2-deficient mice showed increased mortality after challenge with these pathogens.

In the present study, we aimed to understand the role of TRPV2 in cigarette smoke-induced COPD. We demonstrate that TRPV2 plays a key role during phagocytosis in alveolar macrophages. Cigarette smoke exposure significantly decreased TRPV2 expression in alveolar macrophages, both in vitro and in vivo. Furthermore, our data suggest that TRPV2 knockout mice exhibit increased susceptibility to cigarette smoke-induced COPD.

## Methods

### Reagents

The following reagents were used: F4/80 (Santa Cruz Biotechnology, Santa Cruz, CA, USA) and TRPV2 (LifeSpan BioSciences, Seattle, WA, USA) for Immunofluorescence and Western blot. Research cigarettes were purchased from the University of Kentucky. 4′6′-diamidino-2-phenylondole dihydrochloride (DAPI) was obtained from Vector Laboratories (Burlingame, California, USA).

### Mice

TRPV2KO mice were generated as described previously [20]. All primer sequences for genotyping are shown in Additional file 1 All animal experiments were approved by the Gunma University Animal Care and Use Committee, and were performed in accordance with its guidelines (Permit Number; 17–049). Mice were purchased from SLC Japan (Shizuoka). The smoke-exposed emphysema model was induced in 2-month-old female C57BL/6 mice (weight between 16.5 and 20.7 g) by exposure to the smoke of four unfiltered cigarettes per day (3R4F, University of Kentucky), 6 days per week for 2 and 6 months, by using an experimental-smoking apparatus as described previously [20, 21]. Mice tolerated cigarette smoke exposure without evidence of toxicity (carboxyhaemoglobin levels at approximately 10%). All mice were euthanized by cutting the inferior vena cava to induce exsanguination and opening of the chest cavity to induce pneumothorax after inhaled sevoflurane before tissue harvest. This method of euthanasia was performed based on a rule of animal experiment of Gunma University.

### Bronchoalveolar lavage (BAL)

BAL was performed by using a 20 G intravenous catheter inserted into the trachea. Lungs were lavaged four times with 0.75 ml phosphate-buffered saline (PBS), then centrifuged at 3000 × *rpm* for 5 min. Cell pellets were resuspended in 1.0 ml PBS, then used to determine total and differential cell counts with a haemocytometer and cytospins via staining with a Diff-Quick stain kit (Sysmex, Kobe, Japan). Macrophages were prepared by plating the BAL cells on culture dishes overnight and then by removing non-adherent cells. About 95% of the BAL cells were adherent cells. We should be aware that some of the adherent cells may not be macrophages but may be fibroblasts or mesenchymal cells.

### Lung tissue processing

Right lungs were inflated by instillation of 10% formalin at a constant pressure of 25 cm formalin (for 10 min), then fixed for 24 h before paraffin embedding. Serial sections (4-μm thick) were prepared for histologic analysis.

### Cell culture and stimulation

Primary alveolar macrophages were collected from C57BL/6 mice by the BAL method. MH-S cells (derived from mouse alveolar macrophages) were obtained from ATCC. Cells were cultured in RPMI 1640 medium, supplemented with 10% fetal bovine serum and 1% penicillin-streptomycin (Gibco BRL, Gaithersburg, MD, USA) at 37 °C in a 5% CO_2_ atmosphere.

### Morphometry

After fixation, lung sections were stained with haematoxylin and eosin (H&E). Mean linear intercept (Lm; average distance between alveolar walls) was measured by light microscopy, as previously described [20, 21]. Lm is the average distance between alveolar walls and proportional to the amount of pulmonary emphysema. 20 randomly selected representative images were captured from each slide using a motorized OLYMPUS microscope per lung specimen for each mouse. Lm was manually counted from images taken using a winROOF2013. All measurements were performed by a single blinded investigator. The lengths of all the portions on all lines were summed and divided by the total number of alveolar airspace. Airway and vascular structures were excluded from the analysis.

### Immunofluorescence

Immunofluorescence labelling of lung sections was performed by using an antibody against F4/80 (1:100) and TRPV2 (1:250), as previously described [18, 22]. The secondary antibody used for F4/80 and TRPV2 staining was Alexa Fluor 488-conjugated mouse anti-rat IgG (1:100; Invitrogen Life Technologies Corp., Carlsbad, CA, USA). Sections were then washed and mounted using DAPI-containing media to label cell nuclei.

### Phagocytosis assay

Phagocytic function of alveolar macrophages was evaluated by using a FITC-dextran internalization assay. Alveolar macrophages (either MH-S cells or primary alveolar macrophages prepared from mouse BAL fluid) were incubated with 0.35 mg/ml FITC-dextran (M_r_ = 2,000,000; Sigma-Aldrich, St. Louis, MO, USA) at 37 °C for 6 h. Then, cells were washed three times with PBS. Uptake of FITC-dextran by macrophages was assessed by dissolution in RPMI 1640, and the absorbance of the samples at 485–535 nm was determined by using a Denley plate reader.

### Western blot analysis

Cells were washed twice with PBS, harvested, and then lysed in RIPA buffer (20 mM Tris-HCl pH 7.4, 150 mM NaCl, 1% NP-40, 1% sodium deoxycholate, 0.1% sodium dodecyl sulfate (SDS)) containing complete mini and phosSTOP solution (Roche). After sonication and removal of debris by centrifugation, 20 μg protein from each sample was resolved by SDS-polyacrylamide gel electrophoresis and transferred to nitrocellulose membranes. Membranes were immunoblotted with the indicated antibodies: cleaved TRPV2 (1:250) and β-actin (1:500). Antigens were revealed by Immobilon Western HRP Substrate (Millipore, Billerica, MA, USA) after incubation with horseradish peroxidase-conjugated anti-rabbit IgG and visualized by enhanced chemiluminescence (Amersham Biosciences, Buckinghamshire, UK). Band densities were quantified by using Image J software. For in vivo analysis, alveolar macrophages collected by BAL from five mice were pooled because the yield of alveolar macrophages by BAL is very few.

### RNA isolation and qPCR

Total RNA was extracted from the mouse lung tissue and MH-S cells using ISOGEN reagent (Takara Bio, Kyoto, Japan) according to the manufacturer’s protocol. One microgram of RNA was used for reverse transcription with the RNA PCR Kit (Takara Bio) and qPCR analysis was performed using THUNDERBIRD SYBR qPCR Mix (TOYOBO, Osaka, Japan) according to the manufacturers’ protocol. qPCR analysis was carried out using a MX3000P quantitative system (Stratagene, La Jolla, CA). All primer sequences are shown in Additional file 2.

### Statistical analysis

Data are expressed as the mean ± standard deviation. Significant differences were determined by using Student’s t-test and analysis of variance with Tukey–Kramer multiple comparison tests. A *P*-value of < 0.05 was considered significant.

## Results

### TRPV2 expression was reduced by cigarette smoke extract exposure in MH-S cells

**Fig. 1:**
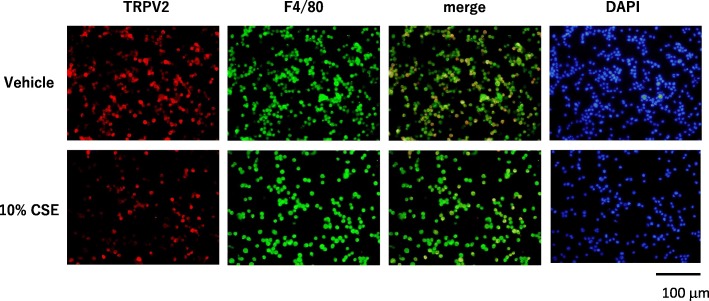
Changes in TRPV2 expression in MH-S cells by immunofluorescence. Immunofluorescent labelling for TRPV2 (red), F4/80 (macrophage, green), merge (yellow), and DAPI (nucleus, blue) in MH-S cells stimulated with vehicle vs 10% cigarette smoke extract (CSE). magnification, × 200; scale bar, 100 μm

**Fig. 2:**
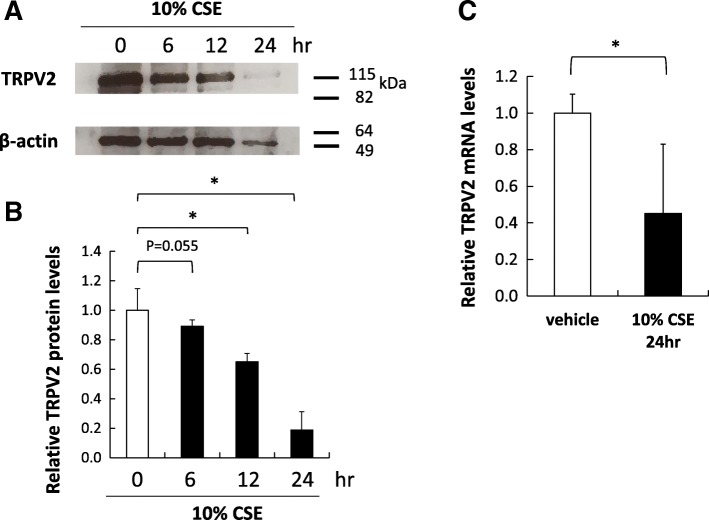
Changes in TRPV2 expression in MH-S cells by Western blot. **a** Representative Western blots of TRPV2 in MH-S cells stimulated with 10% CSE for indicated times. β-actin was used as an internal control. **b** Data represent mean + standard error (*n* = 4) of relative expression level in each sample compared with vehicle standardized for β-actin levels. *p < 0.05

To examine the effects of cigarette smoke extract (CSE) on TRPV2 expression in mouse alveolar macrophages in vitro, we cultured MH-S cells, mouse alveolar macrophages, and performed immunocytochemistry and Western blot analyses on cultured MH-S cells. Immunocytochemistry revealed that the number of the TRPV2-positive cells was reduced after exposure to 10% CSE for 24 h. While 57.5 ± 2.9% of F4/80-positive cells were TRPV2+ in vehicle group, 37.2 ± 3.4% of F4/80-positive cells were TRPV2+ in 10% CSE group (Fig. 1). Protein expression levels of TRPV2 in MH-S cells were evaluated by Western blotting. TRPV2 protein levels, relative to β-actin levels, were decreased in a time-dependent manner and were significantly reduced to ~ 20% of control at 24 h exposure to 10% CSE (Fig. 2a, b). The mRNA and protein expression of TRPV2 in MH-S cells was evaluated by qPCR, and Western blot analysis, respectively. The mRNA levels for TRPV2 were decreased by ~ 60% after exposure to 10% CSE at 24 h (Fig. 2c), while TRPV2 protein expression relative to β-actin was decreased by ~ 80% at 24 h (Fig. 2b).

### Phagocytic function of MH-S cells

**Fig. 3:**
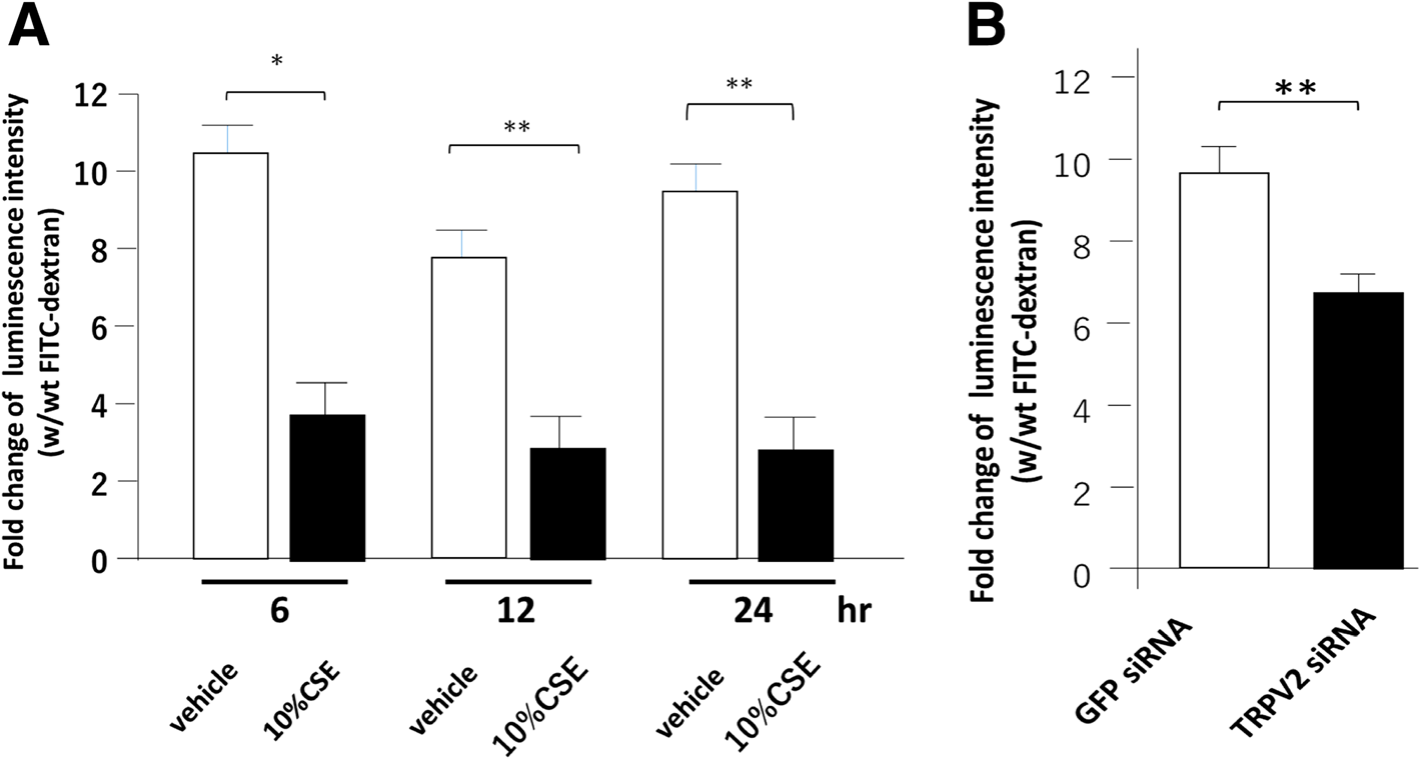
Effects of 10% CSE and TRPV2 knockdown on phagocytosis function. **a** MH-S cells were stimulated with either vehicle or 10% CSE for indicated times, and then exposed to FITC-dextran for 6 h before harvesting for measurement of luminescence intensity. The data represent mean + standard error (n = 4–6) of fold change of luminescence intensity with (w) FITC-dextran vs. without (wt) FITC-dextran. *p < 0.05. **b** MH-S cells transfected with either GFP siRNA (control) or TRPV2 siRNA were cultured with FITC-dextran before harvesting for measuring luminescence intensity. The data represent mean + standard error (n = 4–6 per group) of fold change of luminescence intensity with (w) FITC-dextran vs. without (wt) FITC-dextran. **p < 0.01

We examined the effects of CSE on phagocytic function of MH-S cells by FITC-dextran internalization assay. MH-S cells were incubated with or without 10% CSE for 6, 12, and 24 h; cells were labelled with FITC-dextran for 6 h by adding 0.35 mg/ml FITC-dextran in the culture medium. Internalized FITC-dextran levels in MH-S cells were determined by measuring the absorbance of cell lysates at 485–535 nm; the luminescence intensity of cell lysates prepared from MH-S cells incubated with FITC-dextran was 10-fold higher than that from the cells incubated with vehicle alone. The luminescence intensity of cell lysates prepared from MH-S cells exposed to 10% CSE for 24 h prior to an incubation with FITC-dextran was 3.5-fold higher than those from the cells incubated with vehicle (Additional file 3: Figure S2). Because MH-S cells gave autofluorescence in the absence of FITC-dextran, we expressed the fold-induction of fluorescence intensity of the cells cultured with versus without FITC-dextran (Fig. 3). Importantly, the luminescence intensity was ~ 3.5-fold higher when MH-S cells were exposed to 10% CSE for 6–24 h prior to an incubation with FITC-dextran or vehicle (Fig. 3a). These results suggest that CSE stimulation reduced the phagocytic function of MH-S cells.

### Effect of TRPV2 siRNA on phagocytic activity of MH-S cells

We next examined the effects of silenced TRPV2 expression on phagocytosis. TRPV2 protein levels in MH-S cells transfected with TRPV2 siRNA decreased (Additional file 4: Figure S1A, B). Fold induction of luminescence intensity from internalized FITC-dextran was lower in TRPV2 siRNA-transfected MH-S cells than in siGFP-transfected MH-S cells (6.0-fold vs 10.6-fold, *p* < 0.05) (Fig. 3b). These results suggest that TRPV2 participates in phagocytosis in MH-S cells.

### Phagocytic function of alveolar macrophages collected by bronchoalveolar lavage from cigarette smoke-exposed mice and TRPV2 KO mice

**Fig. 4:**
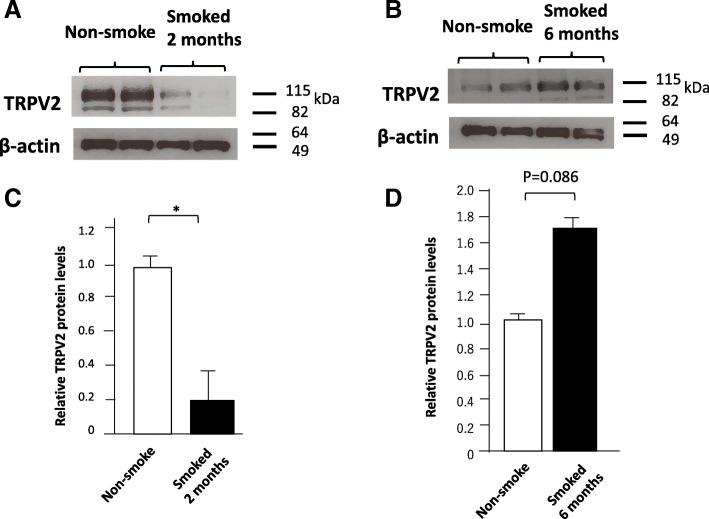
Expression of TRPV2 in alveolar macrophages from bronchoalveolar lavage (BAL) fluid. BAL was performed to collect cells for Western blots. Representative western blots for TRPV2 expression in alveolar macrophages from non-smoke-exposed mice and from mice exposed to smoke for 2 months (**a**) and 6 months (**b**). (**c** and **d**) Bar graphs show the mean + standard error (n = 4–6 mice per group) of TRPV2 protein levels, relative to control values (non-smoked mice) that were set at 1.0. *p < 0.05

We examined the expression of TRPV2 in alveolar macrophage collected by bronchoalveolar lavage (BAL) derived from cigarette smoke-exposed mice (Fig. 4a–d). The expression of TRPV2 was markedly decreased after 2 months smoke exposure in mice (Fig. 4a, c). Interestingly, the expression of TRPV2 was increased after six months smoke exposure when COPD is further progressed compared with 2 months (Fig. 4b, d).

**Fig. 5:**
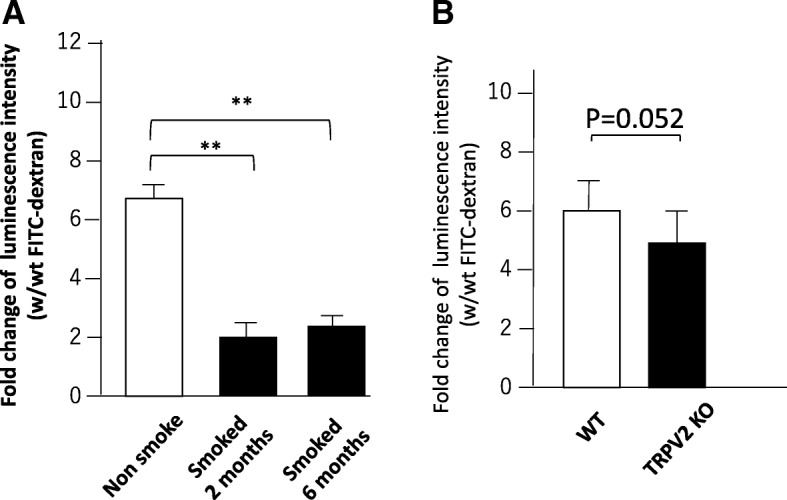
Effects of cigarette smoke on phagocytosis function. **a** Two-month-old C57BL/6 mice were subjected to cigarette smoke exposure for 0 (non-smoke-exposed), 2, or 6 months; then, bronchoalveolar lavage (BAL) was performed to collect alveolar cells from each mouse to examine phagocytosis function. Alveolar macrophages were cultured and exposed to FITC-dextran for 6 h then harvested for measurement of luminescence intensity. The data represent mean + standard error (n = 4–6 per group) of fold change of luminescence intensity with (w) FITC-dextran vs. without (wt) FITC-dextran. **p < 0.01. **b** The phagocytosis function of alveolar macrophage in WT mice and TRPV2 KO mice. 2-monh-old mice were subjected to BAL to collect alveolar macrophages to examine the phagocytosis function. Collected alveolar macrophages were cultured and exposed to FITC-dextran for 6 h, and then harvested for measuring luminescence intensity. The data represent mean + standard error (n = 4–6 per group) of fold change of luminescence intensity with (w) FITC-dextran vs. without (wt) FITC-dextran

We next examined the phagocytosis function of alveolar macrophages from either non-smoke- or cigarette smoke-exposed mice and TRPV2 KO mice at 4 months of age. As shown in Fig. 5 A, alveolar macrophages from non-smoke mice cultured in the presence of FITC-dextran have 6.2-fold higher luminescence luminous intensity relative to those cultured with vehicle; however, this value was reduced to 1.9-fold when alveolar macrophages were prepared from mice exposed to cigarette smoke for 2 months. Consistent with these data, alveolar macrophages derived from mice exposed to cigarette smoke for 6 months showed merely 2-fold greater phagocytosis function, relative to control alveolar macrophages. These results showed that the phagocytic function of alveolar macrophages was reduced by smoking for 2 months, and remained low after 6 months of cigarette smoke exposure. As shown in Fig. 5b, fold induction of luminescence intensity from internalized FITC-dextran was lower in TRPV2KO-derived alveolar macrophages than WT-derived alveolar macrophages (5.9 + 1.3 vs 4.4 + 1.0, *p* = 0.052), suggesting that alveolar macrophages from TRPV2KO mice exhibit weaker phagocytosis than alveolar macrophages from WT mice.

### The susceptibility for emphysema by smoking in TRPV2KO mice

**Fig. 6:**
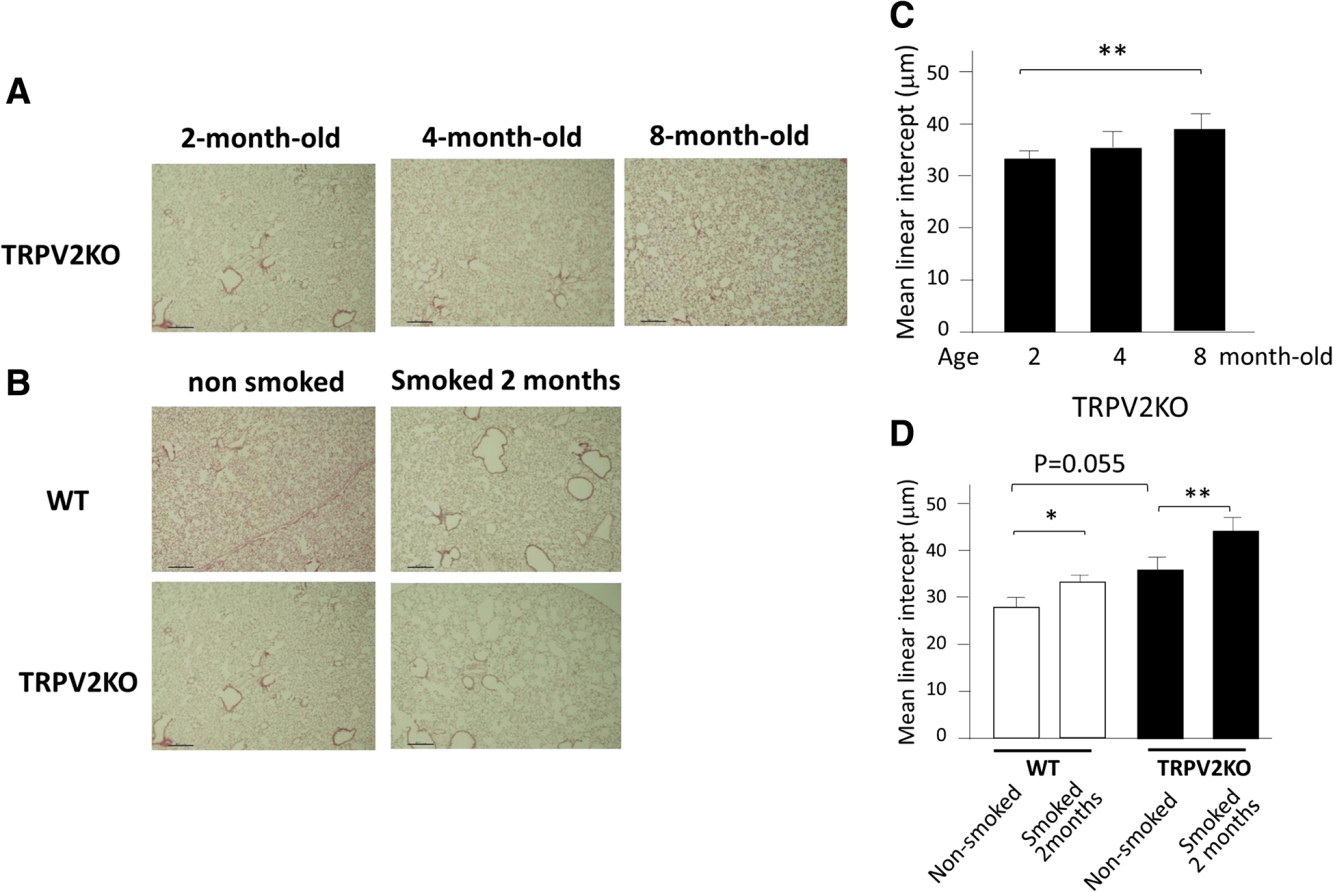
H&E staining of lung specimens in WT and TRPV2KO mice. **a** H&E staining of the lung from TRPV2KO mice of 2, 4, and 8-month-old (magnification, × 100; scale bar, 100 μm). **b** H&E staining of the lung from WT and TRPV2KO mice that were exposed to cigarette smoke for 2 months (magnification, × 100; scale bar, 100 μm). **c** The change of mean linear intercept in TRPV2KO mice. *p < 0.01. **d** The change of mean linear intercept by 2 months cigarette smoke exposure in WT and TRPV2KO mice. *p < 0.05. **p < 0.01. Mean linear intercept (Lm; average distance between alveolar walls) was measured by light microscopy

We examined lung morphological change in TRPV2KO mice and WT mice. An alveolar space gradually enlarges with advancing age in TRPV2KO mice (Fig. 6a), and mean linear intercept was significantly increased in 8-month-old TRPV2KO mice compared to that of 2-month-old TRPV2KO mice (Fig. 6c). Moreover, the emphysematous change was noted in TRPV2 KO mice by 2 months smoke exposure although it was not obvious in WT mice after 2 months-smoke exposure (Fig. 6b). An increase in mean linear intercept in TRPV2 KO mice was more intense than that in WT mice (Fig. 6d). This result shows that alveolar destruction was induced earlier in TRPV2 KO mice than WT mice and suggests that TRPV2KO mice are more susceptible for cigarette smoke-induced emphysema than WT mice.

## Discussion

In the present study, we provided several lines of possibility indicating that the development of cigarette smoke-induced COPD is partly attributed to reduced phagocytosis in alveolar macrophages, mediated by cation channel TRPV2. First, CSE reduced TRPV2 expression and phagocytic function, as assessed by FITC-dextran internalization assays in cultured MH-S cells. Second, TRPV2 knockdown experiments with siRNA showed reduction of phagocytic function. Third, 2 months of cigarette smoke reduced TRPV2 expression in mice. Lastly, alveolar macrophages prepared by BAL from cigarette smoked-exposed mice exhibit reduced phagocytosis, compared with macrophages from non-smoke-exposed mice. These results suggest that TRPV2 in alveolar macrophages serves as a protective channel against cigarette smoke-induced COPD by participating in phagocytosis.

### Suppression of TRPV2 expression in alveolar macrophages by cigarette smoke in vitro and in vivo

Immunocytochemistry of MH-S cells revealed that 10% CSE stimulation reduced the number of TRPV2-positive cells, while the number of F4/F80-positive cells seemed to be less affected by CSE. These results suggest that the CSE-related reduction in TRPV2-positive cells is not due to cell apoptosis. Consistent with this, qPCR and Western blot analyses revealed significant reduction of TRPV2 mRNA and protein levels, respectively, relative to β-actin levels. These findings deserve special attention because it is well documented that cigarette smoke induces oxidative damage, primarily through reactive oxygen species (ROS); ROS has been implicated in apoptosis induction in a variety of cells, including lung epithelial cells, via induction of caspase-3 activity [23, 24]. The molecular mechanisms regulating a decrease in TRPV2 expression in the absence of apoptosis remain to be determined, but based on our finding that TRPV2 mRNA levels were robustly reduced by CSE, we suspect that ROS-mediated pathways that suppress TRPV2 gene transcription or TRPV2 mRNA function may regulate TRPV2 protein levels. Understanding the molecular mechanisms governing suppression of TRPV2 gene expression upon CSE treatment should provide novel insights into the mechanisms of CSE-mediated macrophage dysfunction.

It is notable that, while TRPV2 protein levels were strongly suppressed after 2 months of cigarette smoke exposure, they were restored by 6 months of exposure, when airspace enlargement developed further. The precise mechanisms of increased TRPV2 expression at 6 months of cigarette smoke exposure are unclear, but may involve compensatory mechanisms to prevent the destruction of lung architecture; we have previously reported that collagen type IV and surfactant protein-A (SP-A), which protect against development of COPD, are upregulated despite progression of destructive changes in the lung [25]. Compensatory increases in TRPV2 during COPD progression may underscore the importance of this channel in maintaining alveolar structure.

We prepared macrophages from BAL cells by plating the BAL cells on culture dishes overnight and then by removing the non-adherent cells. We should be aware that this methods may have allowed some non-macrophages to be included in adherent cells. Thus, a decrease in TRPV2 protein in macrophages in BAL cells may be, at least in part, due to a decrease in the number of macrophages in BAL cells rather than the reduced expression of TRPV2 in macrophages.

### The role of phagocytosis by alveolar macrophage in cigarette smoke-induced COPD

Increased oxidative stress and a subsequent chronic inflammatory response are responsible for many deleterious effects of cigarette smoke [26–30]. In addition, increased apoptosis and defective alveolar macrophage phagocytosis contribute to cigarette smoke-induced COPD [13]; several studies have reported a large number of apoptotic cells in the lungs of patients with COPD [31] [32]. Therefore, CSE and cigarette smoke exposure may suppress TRPV2-mediated phagocytosis. TRPV2KO mice have increased susceptibility to cigarette smoke-induced COPD, which suggests that impaired phagocytosis is important in the development of COPD.

### The role of TRPV2 in cigarette smoke-induced COPD

Our finding that reduction of TRPV2 expression increases susceptibility to cigarette smoke-induced COPD is consistent with results reported by Link et al., who showed that TRPV2 plays a critical role in macrophage phagocytosis [18]. Their study revealed that TRPV2-deficient peritoneal macrophages were defective in phagocytosis of a wide variety of phagocytic substrates, such as complement-coated latex beads, zymosan particles, and bacteria. TRPV2 is also required for phagocytic receptor clustering. In our study, we did not examine phagocytic substrates of alveolar macrophages in our cigarette smoke exposure mouse model. However, cigarette smoke contains a complex mixture of > 5000 chemicals, including ROS and carbonyl compounds, which directly injure lung epithelial surfaces [33]. Many apoptotic cells including endothelial cells, alveolar epithelial cells, and inflammatory cells, accumulate in lung tissues of COPD patients [34]. Therefore, in our cigarette smoke exposure mouse model, apoptotic cells are likely to serve as a major substrate for macrophage phagocytosis, also known as efferocytosis. Further studies are necessary to test whether alveolar macrophages utilize signalling through TRPV2 channels to cluster apoptotic cell recognition receptors and activate subsequent intracellular mechanisms that can change the phenotype and function of alveolar macrophages, given that recognition receptors for apoptotic cells may be distinct from those for microbial particles [35].

Our finding that TRPV2-mediated phagocytosis is impaired in smoke-exposure mice suggests that TRPV2 agonists may have potential to ameliorate cigarette-induced COPD in humans. It is intriguing to speculate that probenecid, the prototypical uricosuric agent which has been reported to activate TRPV2 [36], may have therapeutic potential for COPD. This possibility warrants thorough investigation in future studies.

## Conclusion

The present study demonstrated that alveolar macrophages, in which TRPV2 is genetically disrupted or silenced by siRNA, exhibit defective phagocytic function. Together, these results suggest that TRPV2 may be a potential target for the treatment of cigarette smoke-induced COPD.

## Additional files

Additional file 1: Generation of TRPV2KO mice. (DOCX 12 kb)

Additional file 2: Construction of siRNA oligonucleotide and transfection. (DOCX 12 kb)

Additional file 3: **Figure S2.** Comparison of fold changes of luminescence intensity between vehicle- and 10%CSE-exposed MH-S cells in the absence of FITC-dextran. The bars represents mean + SE (n = 4–6) of fold changes of luminescence intensity of cell lysates prepared from MH-S cells exposed to either vehicle or 10% CSE. for 24 h. While luminescence intensity increased 1.5-fold in vehicle-exposed cells, 3.7-fold induction of luminescence intensity was observed in 10%CSE-exposed cells. (PPTX 43 kb)

Additional file 4: **Figure S1.** TRPV2 knockdown by siRNA. (A) MH-S cells were transfected with either green fluorescent protein (GFP) siRNA (control) or TRPV2 siRNA, and whole-cell extracts were prepared for Western blotting for TRPV2 and β-actin. (B) The bars represent mean + SE (*n* = 4–6) of TRPV2 protein levels relative to β-actin levels. Mean value of TRPV2 protein levels relative to β-actin levels in GFP siRNA lanes was set at 1.0. *p < 0.05. (PPTX 119 kb)

## Abbreviations

BAL: Bronchoalveolar lavage
COPD: Chronic obstructive pulmonary disease
CSE: Cigarette smoke extract
H&E: Haematoxylin and eosin
KO: Knockout
SP-A: Surfactant protein-A
TRPV2: Transient receptor potential cation channel subfamily V member 2
WT: Wild-type
