# Supplementary material for: Reduced transient receptor potential vanilloid 2 expression in alveolar macrophages causes COPD in mice through impaired phagocytic activity
Source: BMC Pulm Med. 2019 Mar 26;19:70. doi: 10.1186/s12890-019-0821-y (PMC6434859; doi:10.1186/s12890-019-0821-y)
Supplement: Supplementary file 1 — Generation of TRPV2KO mice. (DOCX 12 kb) [file 12890_2019_821_MOESM1_ESM.docx]

Genotyping was performed via PCR with the following primers: forward primer 1, 5’-CTCCATCTCCACAGAAGTTTCAGCGATA-3’, reverse primer 1, 5’- AGTTCTCAGGGTCCACATTGCCTCAGT-3’ on exon 3, with a PCR product of 562 bp (wild-type), and forward primer 2, 5’-GATGTGTGTAGACGCCAGAAGTAGATGTGG-3’, reverse primer 2, 5’-TGTTAAAAGCAGAACCCAGGGGCATAAGGCAA-3’, with a PCR product of 632 bp (KO).
