# Supplementary material for: Reduced transient receptor potential vanilloid 2 expression in alveolar macrophages causes COPD in mice through impaired phagocytic activity
Source: BMC Pulm Med. 2019 Mar 26;19:70. doi: 10.1186/s12890-019-0821-y (PMC6434859; doi:10.1186/s12890-019-0821-y)
Supplement: Supplementary file 2 — Construction of siRNA oligonucleotide and transfection. (DOCX 12 kb) [file 12890_2019_821_MOESM2_ESM.docx]

Target sequences of mouse siTRPV2 and control siRNA were as follows: siRNA oligonucleotides (mouse siTRPV2: 5’-GTG TGC TGG AAG CCA CGT GTA-3’ (TAKARA Bio, Shiga, Japan) and siGFP: 50-GUU CAG CGU GUC CGG CGA GTT-30 (BONAC Corporation, Fukuoka, Japan). siRNA plasmid transfection was performed with Lipofectamine RNAiMAX Reagent (Invitrogen Life Technologies Corp.), following the manufacturer’s protocol.
