# Supplementary material for: Reduced transient receptor potential vanilloid 2 expression in alveolar macrophages causes COPD in mice through impaired phagocytic activity
Source: BMC Pulm Med. 2019 Mar 26;19:70. doi: 10.1186/s12890-019-0821-y (PMC6434859; doi:10.1186/s12890-019-0821-y)
Supplement: Supplementary file 3 — Figure S2. Comparison of fold changes of luminescence intensity between vehicle- and 10%CSE-exposed MH-S cells in the absence of FITC-dextran. The bars represents mean + SE (n = 4–6) of fold changes of luminescence intensity of cell lysates prepared from MH-S cells exposed to either vehicle or 10% CSE. for 24 h. While luminescence intensity increased 1.5-fold in vehicle-exposed cells, 3.7-fold induction of luminescence intensity was observed in 10%CSE-exposed cells. (PPTX 43 kb) [file 12890_2019_821_MOESM3_ESM.pptx]

## Slide 1
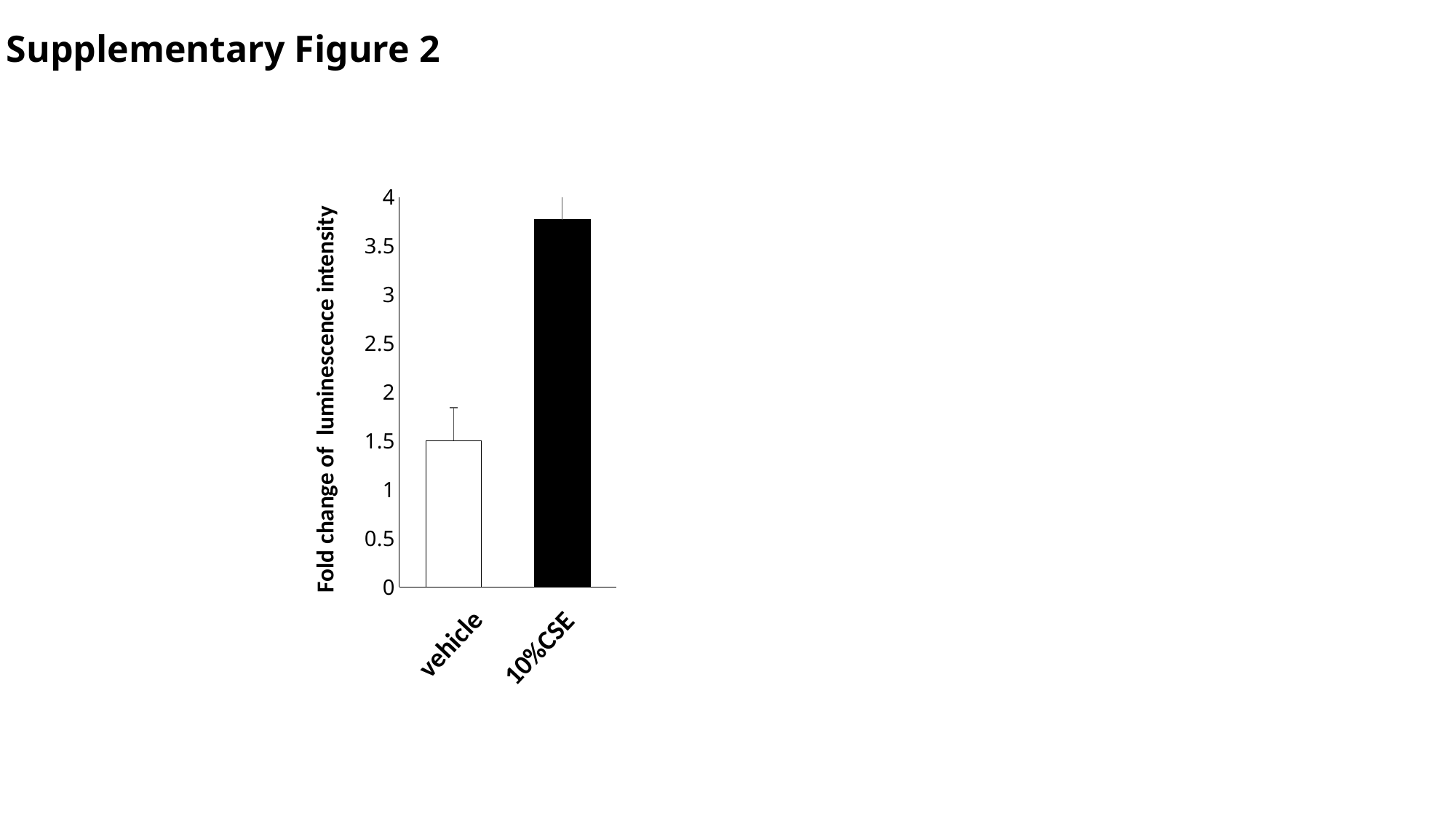

Supplementary Figure 2
### Chart
| Category | |
|---|---|
| vehicle | 1.5006395047987262 |
| 10%CSE 24h | 3.76819659646759 |Fold change of luminescence intensity
10%CSE
vehicle
