# Supplementary material for: Reduced transient receptor potential vanilloid 2 expression in alveolar macrophages causes COPD in mice through impaired phagocytic activity
Source: BMC Pulm Med. 2019 Mar 26;19:70. doi: 10.1186/s12890-019-0821-y (PMC6434859; doi:10.1186/s12890-019-0821-y)
Supplement: Supplementary file 4 — Figure S1. TRPV2 knockdown by siRNA. (A) MH-S cells were transfected with either green fluorescent protein (GFP) siRNA (control) or TRPV2 siRNA, and whole-cell extracts were prepared for Western blotting for TRPV2 and β-actin. (B) The bars represent mean + SE (n = 4–6) of TRPV2 protein levels relative to β-actin levels. Mean value of TRPV2 protein levels relative to β-actin levels in GFP siRNA lanes was set at 1.0. *p < 0.05. (PPTX 119 kb) [file 12890_2019_821_MOESM4_ESM.pptx]

## Slide 1
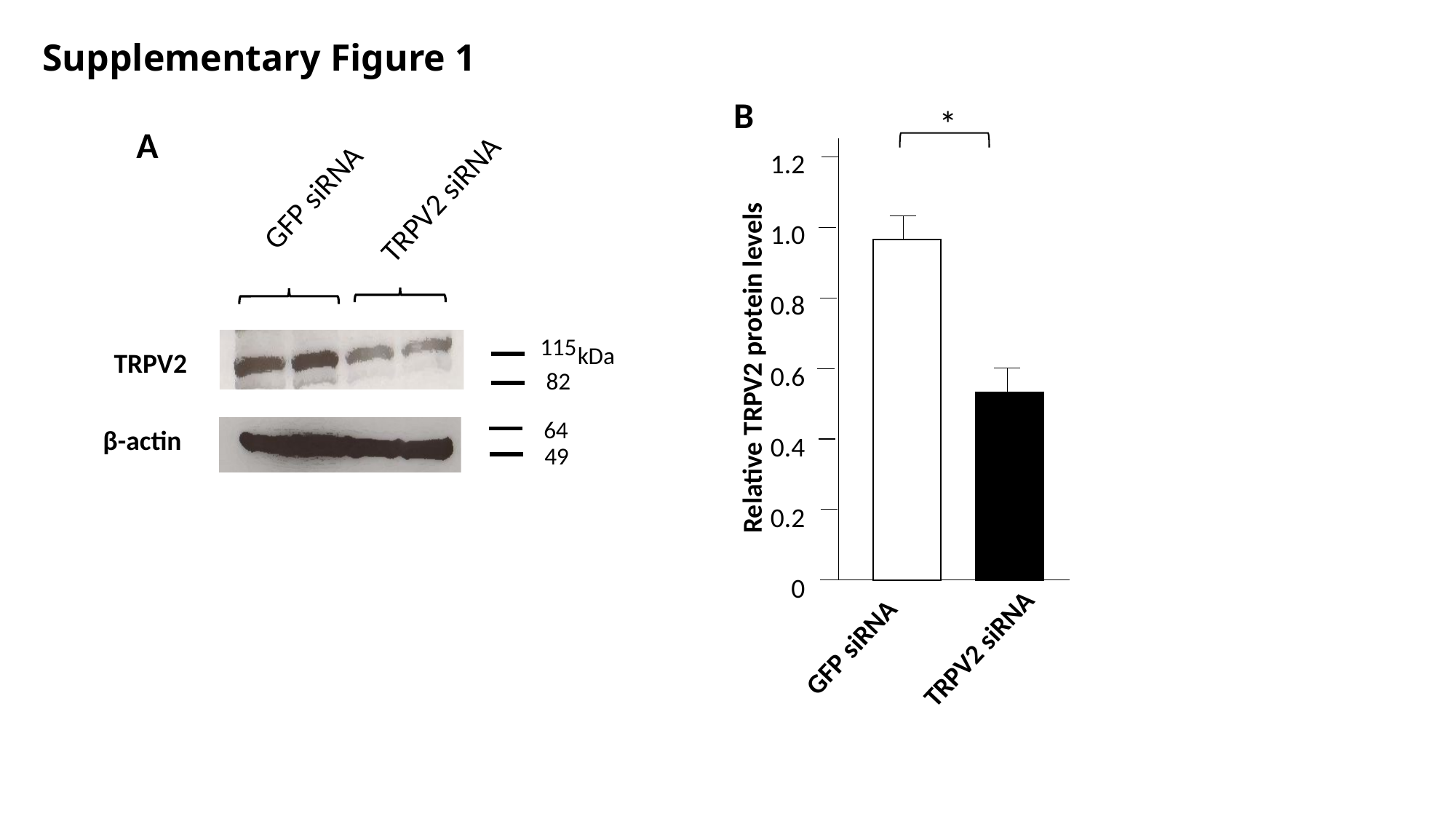

Supplementary Figure 1
B
*
A
1.2
1.0
0.8
0.6
0.4
0.2
0
GFP siRNA
TRPV2 siRNA
115
kDa
TRPV2
Relative TRPV2 protein levels
82
64
β-actin
49
GFP siRNA
TRPV2 siRNA
